# Phenotypic and yield responses of common bean (Phaseolus vulgaris l.) varieties to different soil moisture levels

**DOI:** 10.1186/s12870-024-04856-5

**Published:** 2024-04-04

**Authors:** Refisa Jebessa Geleta, Amsalu Gobena Roro, Meseret Tesema Terfa

**Affiliations:** 1 Ambo University, Department of Horticulture, P.O. Box 19, Ambo, Ethiopia; 2 School of Plant and Horticultural Sciences, Hawassa University, P.O. Box 05, Hawassa, Ethiopia

**Keywords:** Moisture Deficit, Drought stress, Morphological plasticity, Waterlogging stress, Yield component

## Abstract

**Background:**

Morphological plasticity is one of the capacities of plants to modify their morphological appearance in response to external stimuli. A plant’s morphology and physiology are constantly tuned to its variable surroundings by complex interactions between environmental stimuli and internal signals.

In most of plant species,, such phenotypic and physiological expression varies among different varieties based on their levels of particular environmental stress conditions. However, the morphological and yield responses of common bean varieties to different environmental conditions are not well known. The purpose of the study was to evaluate morphological and yield response of common bean to soil moisture stress and to investigate the morphological mechanism by which common bean varieties tolerate fluctuations in moisture stress.

**Methods:**

A pot experiment was carried out to investigate the effects of different moisture levels on the phenotypic and yield responses of common bean varieties. A factorial combination of five common bean varieties (Hirna, kufanzik, Awash-1, Ado, and Chercher) and three moisture levels (control, waterlogging stress, and moisture deficit stress) was used in three replications. Moisture stress treatments were started 20 days after planting, at the trifoliate growth stage. To evaluate the response of each variety, morphological and yield data were collected at week intervals.

**Main results:**

The results indicated that moisture levels and varieties had a significant influence on all growth parameters. Crop phenology was significantly influenced by the interaction effect of moisture level and variety. Exposing Hirna variety to moisture stress led to extended flowering and pod setting by 23 and 24 days, respectively, compared to the other treatments. The results showed that the phenotypic responses to moisture deficit and waterlogging stress varied between varieties. Waterlogging stress had a stronger reduction effect on the fresh weight, dry weight and leaf area of common bean varieties than moisture deficit and the control. Pods per plant, seeds per plant, grain yield per plant, and harvest index were significantly influenced by the varieties, moisture stress levels and their interaction. Except for Chercher and Hirna. However, varieties Ado, kufanzik and Awasha-1 did not show significant differences on the time of flower initiation due to moisture level. Biomass and growth in leaf fresh weight, leaf dry weight, leaf area, leaf number and plant height were significantly influenced by moisture level. When moisture deficit and waterlogging stress occurred, Ado and Awash-1 were more responsive to moisture stress than Hirna, Chercher, and Kufanzik.

**Conclusion:**

Hence, Hirna and Kufanzik varieties were found to be tolerant because they produced higher yields than the Chercher, Awash-1, and Ado varieties.

## Background

Common bean (Phaseolus vulgaris L.) is an herbaceous annual plant grown worldwide for its edible dry seeds or green immature pods. The crop is a highly polymorphic warm-season crop that has two growth habits: erect herbaceous bushes (determinate) up to 20 to 60 cm high and climbing vines (indeterminate) up to 2 to 5 m long [1]. It grows in different agroecosystems [2] and it is a fast-expanding legume crop that provides an essential part of the daily diet and foreign earnings in developing countries [3]. The crop grows well between 1400 and 2000 m above sea level. Although bean is suitable for food security due to its short growing cycle (2.5 to 3 months) and adaptability to different cropping systems [4], because of limited research in terms of genetic improvement, the crop is sensitive to climate change. Climate change-induced moisture stress, diseases, and insect pests pose significant challenges to its productivity [5]. As reported by [6] that moisture stress-induced reduction in stomatal density could decrease stomatal conductance in plants, thereby reducing CO_2_ diffusion and lowering the photosynthetic rate [7] and reducing grain yield. Changes in plant morphology and root structure reduce water and nutrient uptake and finally reduce grain yield [8, 9]. Although water is chemically important, its physical qualities and quantity have an effect on free gas exchange, leading to poor quality and less yield and finally being killed when totally submerged [10]. Optimum water is required for normal plant growth and proper function of the plant organ. Plants exposed to waterlogging could have limited energy metabolism and restricted growth and developmental processes. For this reason, plants respond to waterlogging stress by regulating their morphological structure, energy metabolism, endogenous hormone biosynthesis, and signaling processes [11]. Moreover, waterlogging plants have higher root respiration and cause permanent cell death in roots due to the accumulation of ethylene, which has a negative impact on root and shoot growth morphology, physiology, and yield.

Similarly, plants exposed to drought have reduced rates of growth, cell division, and cell expansion as well as reduced leaf thickness, palisade tissue and spongy tissue during the growth period. The potential of plants to adapt to drought has a direct relation with the water-holding capacity of plants in leaves and tissues under moisture deficit conditions. Plants with higher leaf thickness, palisade tissue and spongy tissue have maximum adaptation potential to drought stress [12]. However, under stress growth conditions, various morphological and physiological adjustments have been observed in different plant species as a coping mechanism. Plants with moisture stress tolerance ability may have water stress avoidance mechanisms, which sustain important physiological processes, such as stomatal regulation and enduring severe dehydration via osmotic adjustment and osmo-protectants and changes in cell size and expansion. However, the degree to which plants respond to moisture stress varies based on the genotypes and the time that plants are subjected to moisture stress. Under severe water deficit conditions, significant changes in shoot and root morphology could be observed, and decreases in growth morphology were more predominant in plants subjected to stress for longer periods. However, the morphological development plasticity of common bean varieties to changing moisture levels has not been well addressed. Therefore, this study was focused on investigating the impact of different levels of soil moisture stress on the morphological and yield components of common bean varieties and studying the morphological mechanism by which common bean varieties tolerate fluctuations in moisture stress.

## Results

### Effect of moisture stress and variety on leaf temperature

Leaf temperature was significantly influenced by variety and moisture level, but the interaction between variety and moisture level did not show difference on the level of leaf temperature on all varieties (Table 1).

**Table 1:** Growth attributes of five common bean varieties as influenced by different soil moisture levels under a shade house, 2019

Varieties	Leaf fresh weight (g) Per plant	Leaf dry weight (g) Per plant	Leaf Area (cm^2^) per plant	Internode length (cm)	Shoot dry weight	root dry weight	Shoot to root ratio
Chercher	7.62^b^	1.68^c^	481.82	2.61^d^	7.23c	1.43	5.09d
Ado	8.81^b^	1.99^abc^	473.15	6.06^c^	8.42b	1.20	6.92bc
Kufanzik	8.61^b^	1.81^bc^	536.67	9.72^b^	8.22bc	1.27	6.49 cd
Awash-1	11.54^a^	2.16^a^	587.67	12.90^a^	11.15a	1.31	8.68a
Hirna	10.92^a^	2.10^a^	557.27	5.92^c^	10.53a	1.38	8.10ab
LSD(0.05)	1.56	0.37	115.24	1.42	1.22	0.27	1.71
SE	0.77	0.18	56.824	0.70	0.60	0.13	0.84
Moisture level							
Moisture deficit	9.20^b^	1.79^b^	485.89^b^	6.24^b^	8.81b	1.24b	7.42b
Waterlogging	6.36^c^	1.42^c^	392.00^c^	6.90^b^	12.54a	1.45a	8.89a
Control	12.93^a^	2.64^a^	704.06^a^	9.18^a^	5.97c	1.26ab	4.86c
LSD (0.05)	1.21	0.28	89.26	1.10	0.95	0.20	1.32
CV (%)	17.21	19.83	22.86	19.98	14.11	21.40	25.35
SE	0.59	0.14	44.01	0.54	0.46	0.10	0.65
Interaction	Ns	ns	ns	ns	ns	ns	ns

Means sharing the same letter in a column of treatment are not statistically significant at the 95% confidence level based on the least significance difference (LSD) comparison method

*CV* coefficient of variance

Among all tested varieties, Hirna variety had 0.53℃ higher leaf temperature than the Ado variety. But no difference between Awash-1, Kufanzik and cherecher variety in terms of leaf temperature. Result also showed that plant exposed to moisture deficit and waterlogging condition significantly raised the leaf temperature by approximately 0.84 °C and 0.32 °C, respectively, compared to the optimal moisture level (control) (Table 2).

**Table 2:** Effect of genotype and moisture level on leaf temperature. The temperature was recorded during the middle of the day (12:00 pm)

**Variety**	Leaf Temperature (℃)
Hirna	23.19a
Awash-1	22.93ab
Chercher	22.88ab
Kufanzik	22.81ab
Ado	22.66b
LSD(0.05)	0.26
SE	0.127
**Moisture Stress**	**Leaf Temperature (℃)**
Moisture deficit	23.28a
Waterlogging	22.96b
Control	22.44c
CV (%)	1.18
SE	0.09
**Interaction**	**Leaf Temperature (℃)**
Variety^*^ Moisture Stress	NS

### Effect of moisture stress and variety on leaf relative water content

The leaf relative water content was significantly influenced due to the main effect of moisture level. However, variety and the interaction effect did not show significant differences between treatments on relative water content of common bean varieties (Table 3). Plants grown under Moisture deficit and waterlogging growth condition reduced Leaf Relative Water Content (LRWC) by 15.9% and 8.72%, respectively as compared to optimum moisture level (Control) (Table 4). However, in terms of varieties, significance difference was not observed in Leaf relative water content.

**Table 3:** Mean value of yield and yield components of five common bean varieties as influenced by different soil moisture levels under a shade house, September 2019 to December 2019

Varieties	Pods per plant	Seeds per plant	Seeds per pod	Grain yield per plant (g)
Chercher	4.66^a^	15.21^a^	3.25abc	3.98^c^
Ado	3.42^b^	11.80^b^	2.98bc	4.14^c^
Kufanzik	3.32^b^	10.27^b^	3.68a	5.25^ab^
Awash-1	3.53^b^	10.50^b^	2.92c	4.43^bc^
Hirna	4.19^ab^	12.99^ab^	3.46ab	5.45^a^
**Mean**	3.824	12.154	3.258	4.65
LSD (0.05)	0.94	2.95	0.60	0.98
SE	0.462	1.455	0.297	0.485
**Moisture level**				
Moisture deficit	2.47^c^	6.96^c^	3.06b	2.09^c^
waterlogging	3.34^b^	10.28^b^	3.13b	4.43^b^
Control	5.66^a^	19.22^a^	3.57a	7.44^a^
**Mean**	3.82	12.15	3.25	4.65
LSD (0.05)	0.73	2.28	0.46	0.76
CV (%)	25.63	25.39	19.32	22.12
SE	0.358	1.127	0.230	0.376

Means sharing the same letter in a column of treatment are not statistically significant at the 95% confidence level based on the least significance difference (LSD) comparison method

*CV* coefficient of variance

**Table 4:** Leaf relative water content of common bean varieties grown under different soil moisture levels

**Varieties**	**LRWC (%)**
Chercher	77.20
Ado	77.57
Kufanzik	74.18
Awash-1	73.21
Hirna	78.15
**Mean**	76.04
**LSD (0.05)**	**Ns**
**SE**	**3.56**
**Moisture level**	**LRWC (%)**
Moisture deficit	68.37c
waterlogging	75.55b
Control	84.27a
**Mean**	76.06
**LSD (0.05)**	**5.6520**
**CV (%)**	**9.93**
**SE**	**2.75**
**Interaction**	**LRWC (%)**
Varieties^*^ Moisture level	**NS**

Means sharing the same letter in a column of treatment are not statistically significant at the 95% confidence level based on the least significance difference (LSD) comparison method

*CV* coefficient of variation

### Effect of moisture stress and variety on phenology of the crops

#### Effect of moisture stress and variety on days required for flowering

The number of days required to reach 50% flowering was significantly affected by the interaction between moisture levels and varieties (Table 5). Kufanzik and Ado varieties were found earlier in flowering time by two to 10 days as compared to the tested varieties, however, change in moisture stress level from optimal to moisture stress level did not affect the time required to flower in both varieties regardless of variety. Suggesting that both varieties may have wider moisture adaptation potential than the rest of varieties. Hirna variety grown under moisture deficit and water logging conditions significantly delayed its flowering time by 9–23 days under changing moisture level as compared to the others tested varieties. In this study it was observed that, the variety which took longer time for flowering could also extended its pod formation. However, the effect was stronger in Awash -1 and Hirna under moisture deficit growth conditions (Table 5). Moreover, exposing the Chercher variety to moisture deficit and waterlogging extended the flowering time by two days compared to the control. In this study, it was observed that Kufanzik and Ado varieties were found stable in phenological stage and time of flowering under changing moisture level than the tested varieties.

**Table 5:** Effect of different levels of soil moisture on days to flowering, days to pod formation and days to physiological maturity of common bean varieties under a shade house, 2019

Moisture level	Variety	Days to flowering	Days to pod formation	Days to physiological maturity
**Moisture deficit**	Chercher	44b	49c	85bc
	Ado	32 g	39f	85bc
	Kufanzik	30 h	39f	85bc
	Awash-1	39e	55b	85bc
	Hirna	53a	63a	84c
**Waterlogging**	Chercher	44b	49c	85bc
	Ado	32 g	39f	89a
	Kufanzik	30 h	39f	89a
	Awash-1	35f	41e	87ab
	Hirna	41cd	44d	87ab
**Control**	Chercher	42c	49c	83c
	Kufanzik	30 h	39f	85bc
	Awash-1	35f	40ef	87ab
	Hirna	40de	44d	85bc
**LSD (0.05)**		1.29	1.26	2.35
**CV%**		2.08	1.69	1.62
**SE**		0.6325	0.6164	1.1473

Means sharing the same letter in a column of treatment are not statistically significant at the 95% confidence level based on the least significance difference (LSD) comparison method

*CV* coefficient of variance

### Effect of moisture stress and variety on days to pod formation

The analysis of variance showed that day to pod formation was significantly influenced by the interaction effect of varieties and moisture levels (Table 5). The Kufanzik variety took the shortest days to pod formation irrespective of moisture levels, while the Hirna variety subjected to drought stress took the longest days to pod formation. However, the change in moisture level did not significantly influence the days required for pod formation in the Chercher, Kufanzik and Ado varieties (Table 5), whereas Awash-1 and Hirna were responsive to the days required for pod formation under changing moisture.

### Effect of moisture stress and variety on days to physiological maturity

Physiological maturity of the crop is the time when dry matter accumulation in the seeds or edible part of the plant is stopped and when maximum growth occurs. In this study, it was observed that the days required to reach the maximum physiological maturity were significantly (*P* < 0.01) influenced by the interaction effect of moisture levels and varieties (Table 5). Earlier days to physiological maturity were recorded from all varieties except under waterlogging condition. Waterlogging significantly delayed the total days required to reach the stage of physiological maturity by 2–6 days in Ado, Kufanzik, Awash-1 and Hirna compared to genotypes exposed to control and moisture stress. (Table 5). In general, the days to physiological maturity were 0.6 days and 2.6 days earlier in the Hirna and Chercher varieties, respectively, as a result of drought and waterlogging stresses compared to the control. It is suggested that environmental stress significantly influenced the time when dry matter accumulation in the common seed variety ceased (Table 5).

### Phenotypic response of common bean

#### Effect of moisture stress and variety on fresh and dry weight of leaves

The fresh and dry weights of leaves were significantly (*P* < 0.01) influenced by the main effect of variety and moisture levels, but no significance difference between treatments due to the combined effect of variety and moisture levels (Table 6). Among the measured varieties, the maximum leaf fresh weight and dry weight were recorded from the Awash-1 and Hirna varieties, and the minimum leaf fresh weight was measured from the Chercher (7.62gm), Ado (8.81gm) and kufanzik (8.61gm) varieties (Table 6). The highest leaf dry weight was also recorded from Awash-1 and Hirna, while the lowest leaf dry weight was measured from the Chercher (1.6gm) variety (Table 6). Regarding moisture levels, the optimum moisture level (control) produced the highest leaf fresh weight (12.93gm^)^ and leaf dry weight (2.64gm) compared with plants exposed to waterlogging and drought stress growth conditions. Under optimum moisture level (control) growth conditio ns, the maximum leaf fresh weight (10.92 gm) and dry weight (2.10 gm)-producing varieties (Hirna) were able to give the highest grain yield per plant.

**Table 6:** Description of the experimental treatments

Moisture levels	Varieties	Description of treatments
Control	Chercher Ado Kufanzik Awash-1 Hirna	The pots in the treatment were watered daily to control the soil moisture at optimum
Waterlogging stress	Chercher Ado Kufanzik Awash-1 Hirna	Each pot was placed on a saucer (bowl) to hold the drained water through the bottom of the pot, and the water was maintained at 2 to 3 cm above the soil throughout the experimental period (Flooded)
Moisture deficit	Chercher Ado Kufanzik Awash-1 Hirna	Pots were watered within the interval of five days at the first wilting appearance to control the treatment to wilting stress

### Effect of moisture stress and variety on leaf area and internode length

Leaf area was significantly (*P* < 0.01) influenced by moisture levels, but the main effect of varieties and the interaction effect did not show significantly difference between treatments in terms of leaf area (Table 1).

Plants exposed to waterlogging treatment and moisture deficit growth condition decreased the leaf area by 312 cm2 and 218.1 cm^2^, respectively, compared to plants grown under the optimal moisture level (control) (704.06 cm2). The result in the change in the leaf traits, such as leaf area, change along moisture gradients help in predicting how plants will cope with future global climatic changes.

Similarly result indicated that, change in moisture level significantly influenced the internode length among treatments. However, the interaction effect of both factors did not show a significant (*P* > 0.05) difference in internode length (Table 1). Awash-1 had the longest internode length, followed by the Kufanzik variety, but a shorter internode length was recorded with Chercher. This difference might be related to the growth habit of the genotypes, in which the Awash-1 and Kufanzik varieties are characterized by climbing growth habits compared to the other varieties measured. However, the interaction between genotype and moisture stress level did not significantly affect the internode length.

Plants exposed to moisture stress had a significant reduction in internode length compared to the optimal moisture level; however, there was no significant difference between waterlogging and moisture deficit in terms of internode length. This suggested that the growth habit of the crop also has a strong effect on the regulation of internode length and plant height irrespective of moisture stress conditions.

The root/shoot ratio is the ratio of the belowground biomass to the aboveground biomass, which is the parameter that most directly reflects biomass allocation by plants. In this study it was observed that, shoot dry weight and shoot-to-root ratio were significantly influenced by the main factors. The Awash-1 and Hirna varieties had the highest shoot dry weight and shoot-to-root ratio, while the Chercher varieties had the lowest shoot to root ratio. Similarly, it was observed that plant exposed to moisture deficit and waterlogging had higher shoot to root ratio than plant grown under optimal moisture level (Control). Suggesting that plants with a higher proportion with shoots can collect more light energy, while plant with higher proportion of roots are more advantageous in compete more effectively for soil moisture and nutrients. In relation to root dry weight, there was no significant difference due to varieties, but moisture level significantly increased with pant grown under waterlogging than moisture deficit or optimum moisture level (Table 1).

### Effect of moisture stress and variety on leaf number, plant height and root length

The interaction effect of varieties and moisture levels significantly influenced leaf number, plant height and root length of common bean varieties (Table 7). The highest leaf number was recorded from Awash-1 variety treated with the optimal moisture level (control), whereas the lowest leaf number was recorded from Ado, Kufanzik and Hirna varieties which were subjected to waterlogging and moisture deficit growth condition (Table 8). Plant exposed to waterlogging and moisture deficit growth conditions significantly reduced the number of leaves as compared to the optimum moisture level (control). It was observed that Cherecher, Kufanzik and Awash-1 exposed to waterlogging throughout the growth period significantly reduced leaf numbers by 24.22, 12 and 32.9, respectively, compared to plants exposed to the control. Similarly, common bean varieties such as Cherecher, Kufanzik, Awash-1 and Hirna exposed to water deficit for five days during the growth period had significantly reduced leaf numbers by 24.2, 12, 35.9 and 17.15 compared to the optimum moisture level (control), respectively. In contrast, flooding the media with excess moisture and exposure to moisture deficit during the growth period did not significantly influence the number of leaves developed with the Ado variety. However, in this study, it was observed that there was no significance difference in leaf area due to changing the moisture level, but significantly maximum reduction in leaf area was recorded under moisture stress growth condition than control (Table 7), suggesting that a change in moisture level had a stronger effect on regulating leaf area than variety.

**Table 7:** Effect of different soil moisture levels on leaf number, plant height and root length of common bean varieties grown in a shade house from September 2019 to December 2019

Moisture level	Variety	Leaf number	Plant height(cm)	Root length(cm)
**Moisture deficit**	Chercher	35.11 cd	27.56 g	35.66bc
	Ado	28.33def	34.44 fg	28.16def
	Kufanzik	27.11def	53.78cde	35.16bc
	Awash-1	38.66c	75.44b	29.66cde
	Hirna	22.44f	36.33 fg	30.16cde
**Waterlogging**	Chercher	35.11 cd	27.56 g	35.66bc
	Ado	28.33def	34.44 fg	28.16def
	Kufanzik	27.11def	53.78cde	35.16bc
	Awash-1	71.55a	101.67a	36.66b
	Hirna	39.55c	52.11cde	36.66b
**Control**	Chercher	59.33b	41.22ef	31.16bcde
	Ado	35.66 cd	56.33 cd	31.50bcd
	Kufanzik	39.11c	92.78a	43.66a
	Awash-1	71.55a	101.67a	36.66b
	Hirna	39.55c	52.11cde	36.66b
**LSD (0.05)**		9.15	13.19	6.38
**CV%**		15.28	14.02	13.30
**SE**		4.468	6.44	3.115

Means sharing the same letter in a column of treatment are not statistically significant at the 95% confidence level based on the least significance difference (LSD) comparison method

*CV* coefficient of variance

**Table 8:** Physical and chemical characteristics of the experimental soils

N^o^	Properties	Obtained Values
1	Sand (%)	76
2	Clay (%)	6
3	Silt (%)	18
4	Class	Sandy loam
5	Organic carbon (%)	4.67
6	Total nitrogen (%)	0.26
7	Available phosphorus (ppm)	13
8	pH-(H2O)	7.54
9	pH-(KCL)	6.62
10	EC(µs)	5.04
11	CEC(MEQ/100gsoil)	35.73
12	FC (%)	32.67
13	PWP (%)	22.1

Similarly, it was observed that plant height was significantly (*P* < 0.01) affected by the interaction effect of varieties and moisture levels (Table 7). The results indicated that plants exposed to drought stress showed a strong reduction in plant height compared to the same genotype grown under optimum moisture conditions. Among the tested varieties, both varieties were grown under optimum moisture conditions. Moreover, all varieties exposed to waterlogging and water deficit (drought stress) did not show significant differences in terms of plant height, except the Awash-1 genotype, which significantly reduced the plant height under water longing and drought stress, in which a strong reduction was observed under drought stress conditions.

In this study, it was also observed that root length was significantly (*P* < 0.01) influenced by the interaction effect of both varieties and moisture levels (Table 7). The highest average root length was recorded from the Kufanzik variety at the optimum moisture level, whereas the Chercher and Ado varieties had the lowest average root length under waterlogging stress growth conditions (Table 7). Moreover, it was observed that the reduction in root length was stronger in varieties exposed to waterlogged medium than in plants grown under optimum moisture level and water deficit growth conditions. Under moisture deficit conditions, the longest root length was observed for the Kufanzik varieties subjected to optimum moisture level (Table 7). Change in moisture level significantly influenced the root length of Kufanzik, Awash-1 and Hirna, however the effect was stronger when moisture stress changed from optimal to moisture stress (Both waterlogging and moisture deficit) condition on Kufanzik variety, suggesting that Kufanzik is more sensitive in regulation of root length under changing moisture environment.

### Yield response under moisture stress growth condition

#### Effect of moisture stress and variety on pods per plant and seeds per plant

The analysis of variance results showed that pods per plant and seeds per plant were significantly (*P* < 0.01) influenced by the main effect of varieties and moisture levels but not influenced by the interaction effect of both varieties and moisture levels (Table 3). For both parameters, Chercher and Hirna varieties produced the maximum number of pods plant^−1^ and seeds plant^−1^, but Ado, Kufanzik, and Awash-1 similarly accounted both for the minimum number of pods plant^−1^ and seeds plant^−1^ (Table 3). Among the varieties tested, Chercher variety produced a significantly higher number of pods and seeds per plant than the Ado, Kufanzik and Awash-1 varieties (Table 3). This finding indicates that there is an association between the number of pods per plant and the number of seeds per plant, as the highest number of pods per plant resulted in the maximum number of seeds per plant for Chercher. With regard to moisture level, the optimum moisture level performed consistently better than plant growth under waterlogging and moisture deficit stress growth conditions. The highest number of pods per plant and seeds per plant were recorded from the control growing conditions, and a significant reduction was observed from the plant moisture deficit stress condition followed by the waterlogging stress condition.

### Effect of moisture stress and variety on seeds per pod, grain yield and harvest index

The analysis of variance revealed that significant (*P* < 0.01) differences were observed among treatments in the main effects of variety and moisture level, but the interaction effect did not show statistically differences among the treatments (Table 3). The highest seeds per pod and grain yield were obtained from the varieties Hirna and Kufanzik, while the lowest was obtained from the varieties Chercher, Ado, and Awash-1 (Table 3).

Among all varieties tested, it was found that Hirna variety has large seed size but shows few pods, which might be due to its inherent genetic potential. In the case of soil moisture, the effects of moisture levels on grain yield were significantly variable. Plants exposed to optimum moisture levels had a positive effect on yield because optimum moisture levels gave better yields compared to plants exposed to waterlogging and moisture deficit stress conditions (Table 3).

**Fig. 1:**
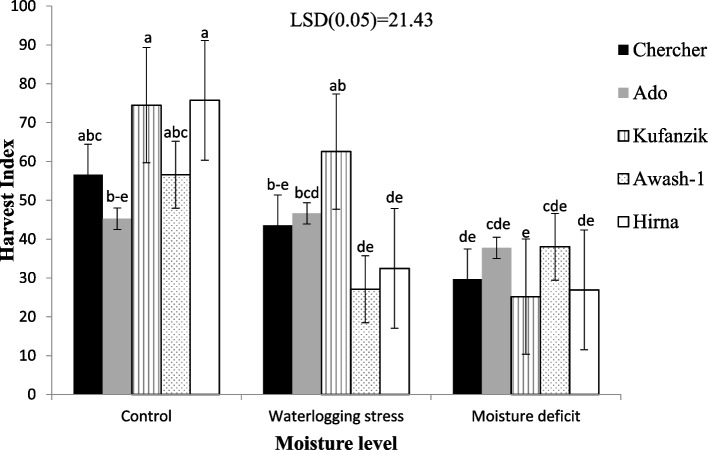
Mean value of the harvest index of five common bean varieties as influenced by different soil moisture levels grown under a shade house, 2019

The harvest index was significantly influenced by moisture level (*P* < 0.01) and the interaction effect of variety and moisture level (*P* < 0.05). However, there was a non-significant difference among varieties (*P* > 0.05) (Fig. 1). Accordingly, the Hirna, kufanzik, Chercher and Awash-1 varieties grown under optimum moisture levels (control) and the Kufanzik variety subjected to waterlogging stress produced the highest harvest index (HI), whereas the kufanzik variety treated with moisture deficit stress gave the lowest harvest index (Fig. 1). The increased harvest index for the Chercher and Hirna varieties might be due to having the highest grain yield while having the lowest biological yield. The highest yield (Pod, seeds and grain Yield) reduction was recorded from moisture deficit growth conditions followed by waterlogging stress.

## Discussion

### Effect of moisture level on leaf temperature of common bean varieties

It is well known that; leaf temperature is the guarantee for the plant to carry out the life activities and closely related to plants’ healthy growth and crops’ planting management. It is reported that, the optimal leaf temperature range for photosynthesis in many plants is between 15 C and 30 C for normal atmospheric concentrations of CO2. However, the levels of temperature build in leaf vary based on the air humidity and crop type. In this study the higher leaf temperature in Hirna might indicates that Hirna variety takes in more energy than it puts out and that the Ado variety may lose more energy than it gains. As [13] reported, the temperature of a leaf is the result of energy flow between it and its surroundings, and the leaf exchanges energy through the basic physical processes of radiation, conduction, convection, and evaporation. This result is in agreement with the finding of [14] who reported that water-deficit conditions resulted in considerable increases in leaf temperature. Plants that are able to maintain lower leaf temperature and optimum stomatal conductance at higher temperatures are therefore better able to maintain higher leaf water content and lower canopy temperature. As [15] reported that a negative relationship exists between the leaf water content and leaf temperature. It has been suggested that enhanced transpirational cooling may be a useful trait in identifying genotypes with thermal plasticity to adapt to climate change [16].

### Effect of moisture level on leaf relative water content

Water potential as an estimate of the energy status of plant water is useful in dealing with water transport in the soil–plant-atmosphere continuum. Under normal growth condition the LRWC range between 98% in fully turgid transpiring leaves to about 30–40% in severely desiccated and dying leaves, depending on plant species [17]. In our study, although there is no significant differences between varieties did not show turgidity as there value is less than 98%. However, as moisture stress increases due to deficit and waterlogging the LRWC is significantly reduced and the effect was pronounced more under deficit than waterlogging Such variability in the leaf relative water content indicated that, the rise in leaf temperature and the marked reduction in the leaf water content in leaves might be due to the reduction in root uptake capacity as a result of the lack of oxygen caused by waterlogging and absence of root hairs under moisture deficit [18, 19].

### Phenotypic responses under moisture stress condition

#### Effect of moisture level on fresh and dry weight

Environmental stress in many crop plants, which is one of the most severe environmental stresses and affects almost all plant functions. Water stress which causes serious reduction in total dry matter accumulation, growth, quantity, and quality in many plants might be due to reduction in cell division and multiplication. The reduction in leaf fresh and dry weight of common bean varieties in our investigation might be due to low turgor pressure, which suppresses cell expansion and growth in leaves [20]. This finding agrees with the previous findings reported by [17, 21]), who found that the leaf dry weight and yield of common beans decreased when plants were exposed to drought stress. Furthermore, an early morphological response to drought stress is the stress avoidance strategy for the plant through adjustment of plant growth rates and biomass allocation in different plant species [22]. Moreover, the higher the leaf dry mass per unit area (LMA) is considered to be an indication for the variety with higher photosynthetic capacity, which actually implies that foliar water mass (leaf fresh weight minus leaf dry weight) is proportional to leaf dry weight during leaf growth. Plant resistant to moisture stress varies among plant species. Plants, which, have the ability to reduce their resource utilization and adjust their growth to cope against adverse environmental conditions like moisture stress may have strong plasticity to perform under changing climatic conditions.

### Effect of moisture level on leaf area and internode length

Phenotypic plasticity is the ability of an organism to express different phenotypes in different environments. Moisture stress, one of the crucial factors for the growth and development of plants, is a highly changing resource in nature, and almost all plants are exposed to a certain degree of moisture stress in their life time. Such fluctuation of moisture stress significantly affects leaf area expansion and plant height. Report from [20, 23] indicated that, drought stress primarily reduced the leaf emergence rate and the leaf area in different plant species. As an escaping mechanism from moisture stress, plants enter the senescence stage to reduce the transpiration area and minimize the loss of water through leaves [24]. The reduction in leaf area leads to a decrease in the source capacity of the canopy and results in reduced photosynthetic capacity [25]. Indeed, loss of leaf area, which could result from smaller younger leaves and inhibition of developing foliage expansion, is thought to be an adaptation mechanism to moisture deficit [26]. Leaf-area plasticity is important to maintain the control of water use in crops. This result is in agreement with the previous report of [27] who stated that determinate plants also have shorter internodes and fewer nodes than indeterminate plants. The change in Leaf size, number, functional traits, and photosynthetic capacity are closely related to plant size and competitive abilities under harsh environmental growth condition. The presence of small leaf area and reduced shoot length is considered as an adaptive strategy to minimize transpiration rate through reduced leaf surface area and regulating the assimilate partitioning toward flowering than shoot elongation.

### Effect of moisture level on leaf number, plant height and root length

In many plants, Vegetative traits can respond directly to changes in the environment, such as those occurring under climate change. That phenotypic plasticity could be an adaptive if the phenotypic changes increase survival and performance in the environment compared with plants that do not express phenotypic changes. In our study, it was observed that modification in leaf number, plant height and root length was induced due to moisture stress and variety. Similar findings were reported by [28], who noted that a low irrigation level reduces the total number of leaves per plant. This might be due to the reduction in plant water status, which reduces shoot elongation, leaf expansion, and inhibition of cell division or cell enlargement. This finding is in line with the results of [29, 30], who reported that plant height was affected by the severe influence of water stress compared with unstressed plants. The observed reduction in plant height was associated with a decline in cell elongation and rapid senescence of leaves under water stress [31].

Furthermore, [32] reported that plant height decreased for mung bean grown under no irrigation and increased with the amount of irrigation. As plants are exposed to different soil moisture levels, it is obvious that the root system is highly influenced based on the availability of soil moisture and genotypes. This suggests that the level of oxygen in the growing medium may play a critical role in regulating root extension and functionality, as it determines the root orientation and the metabolic state of the root [33, 34]. It was clearly stated that plants develop strategies for maintaining turgor by increasing root depth or developing an efficient root system to maximize water uptake and by reducing water loss through reduced stomatal conductance, reduced absorption of radiation, leaf rolling or folding and reduced leaf area [35]. This result is in agreement with a previous report that noted that a greater root length under water deficit conditions contributes to improved drought resistance of the common bean [36]. Under moisture deficit conditions, roots extend their length, increase their surface area, and deplete immobile nutrients [37]. Previous research has also shown that a deep and dense root system in common beans and high root mass [38, 39] correlate with effective water use under moisture deficit conditions.

Deep rooters may have been able to maintain cooler temperatures by accessing deep water reservoirs, allowing them to maintain water potential in adverse conditions. This finding is supported by the previous finding of [40], who stated that the ‘cool’ varieties under water stress showed a deeper root system allowing the extraction of 35% more water from the 30–90 cm soil profile than plants with shallow root systems. As report indicated cellular adaptability vary based on the nature of the plant and environmental stimuli, in which adaptive processes consist of increased cellular size and function, increase in cell number, decrease in cell size and metabolic activity, or a change in the phenotype of the cells [41]. Therefore, the reduction in plant height and leaf area under moisture stress may be associated with the decline in the cell division or change in the phenotype of the cells and cell enlargement.

### Phenological responses under moisture stress condition

#### Effect of moisture level on days required for flowering

The days required for plant to shift from vegetative growth stage to reproductive phase vary based on crop type, phenological stage, amount of phot assimilate accumulated and environmental stress condition. In our study it was observed that common bean variety exposed to different moisture stress level had different days to flower initiation. This suggested that changing moisture stress from an optimal irrigation interval to severely water logged and to moisture deficit conditions significantly influenced the days required to flower for some varieties but not for all types. Such variability in the days required to flower between treatments might be due to differences in the genetic potential of the varieties to tolerate or escape moisture stress.

Many studies have reported that the flowering period in different common bean genotypes varies based on soil moisture [9, 28]. Similar findings were reported by [42] and genotype [43]. These observations suggest that common bean growing in extreme soil moisture levels (water logging and water deficit) delay flowering by one to 13 days depending on the sensitivity of genotypes. Among the tested common bean, Chercher, Awash-1 and Hirna were found to be sensitive to moisture stress, and the sensitivity was stronger in Hirna than in the others. Early flowering under optimum (Control) moisture conditions might be due to the good performance of the root system in absorbing nutrients and moisture that help plants accumulate photosynthesis assimilates in plant tissue. The plant autonomous factor (the amount of carbohydrate accumulated) significantly affects flowering time and induces early flowering, which is one of the stress escape mechanisms for most flowering plants. Such an escape strategy causes the completion of the plant life cycle in advance of the damaging effects of drought [44, 45]. Moisture stress has the potential to cause plastic or evolutionary changes through avoidance or escape strategies in plants. Different reports have indicated that, with plasticity, the expression of the phenotype is shaped by environmental conditions [46].

### Effect of moisture level on pod formation and development

The pod formation, development and filling in many of the plant species is determined by the availability of moisture, nutrients, amount of assimilate supplied and the balance of endogenous plant growth regulators. In this study it was observed that viability in moisture level significantly influenced the pod formation and seed number with in common bean varieties. The results revealed that there was a link between days to pod formation and days to flowering, as earlier days to pod formation were recorded for varieties that flowered earlier. Longer days to pod formation were observed in the Hirna varieties subjected to drought stress. Several studies have indicated that pod formation has no direct relation with the time of pod formation. In some plants, pod formation is very slow after flowering, and in some plants, it is very fast; however, this condition varies from genotype to genotype [47]. In this study, the Hirna variety may be slower than others in initiating flowering and pod development. As [48] reported, individual vegetative growth is limited by the amount of total resources available for changing the size and number of vegetative and reproductive organs. Similarly, morphological plasticity, expressed as a consequence of environmental variations, may also affect the expression of the gene that is responsible for changing the phenology of the plant [49]. Such variability in maturity time could be the way of escaping or tolerating environmental stress in plants. Furthermore, the sensitivity to environmental stress varies from genotype to genotype. A previous report indicated that the timing of phenological events and biomass allocation to different plant organs can be a mechanism for plants to adapt to stressful growth environments, and such phenological plasticity may have important impacts on the overall success of genotypes in particular environmental conditions [50].

### Effect of moisture level on pods per plant and seeds per plant

Moisture Stress at this time transition stage from vegetative to reproductive stage reduces the number of pods per plant as the plants are no longer able to produce new blossoms and pods and this might lead to reduction in total yield. Similarly, the number of seeds per pod and the size of the seed can also be reduced if the moisture stress occurs at seed filling stage.

In line with this finding, [20, 51] reported that drought stress affects yield components such as the number of pods per plant, number of seeds per pod, seed weight and harvest index. Such a reduction in pod number and seed number per pod might be due to a reduction in flower and pod development as a result of moisture stress before flower and pod induction. An early study indicated that the loss of seed yield is maximal when drought occurs during flowering and early pod development [52]. This result is in line with previous findings that, drought stress affects yield components such as the number of pods per plant, the number of seeds per pod, seed weight, and harvest index in different plant species [20, 51]. Furthermore, a reduction in the harvest index was observed as a result of moderate moisture stress in common beans [22]. Thus, traits of possible interest for improving crop tolerance to drought would include a high harvest index [53, 54].

### Effect of moisture level on days to physiological maturity

Previous study indicated that, environmental stress occurring due to climate change significantly reduce plant height, total leaf area, days to physiological maturity, number of grains per plant and grain yield [55]. Similarly, it was observed that crops with early physiological maturity can be one of the mechanisms for reducing the time required for vegetative growth and rapidly transitioning to the reproductive phase before stress causes permanent damage to plant cells. A short growing cycle has been previously recognized as a significant escape mechanism from drought in common beans [26]. Similarly, [56] reported drought tolerance of early-maturing genotypes, given their lower net water requirement throughout their plant life cycle compared with late-maturing genotypes. Days to physiological maturity extended for Ado plants exposed to waterlogging stress exhibit stomatal closure, limited water uptake, oxygen deficiency, and a significant decline in photosynthetic rate [57]. Waterlogging stress is also known to alter physiological mechanisms and have a negative impact on several physiological and biochemical processes in plants due to a lack of essential nutrients such as nitrogen, magnesium, potassium, and calcium [57]. This is due to waterlogging stress, which reduces nutrient and solute transport across the plant.

## Yield responses under moisture stress condition

### Effect of moisture level on seeds per pod, grain yield and harvest index

Under changing climatic condition moisture availability is one of the major factor affecting crop productivity. The reduction in grain yield under moisture stress conditions might be attributed to flower abscission and embryo abortion when the plants were exposed to extended drought stress at the flowering and pod filling growth stages. It was reported that post flowering water stress caused yield losses of up to 50% due to reduced seed filling duration [58, 59]. Moreover, water stress during the flowering and pod filling periods reduced seed yield and seed weight and accelerated the maturity of dry bean [25]. The main criterion for selecting cultivars tolerant to low water availability in crops such as the common bean, where the product of interest is grain, is related to the characteristics that result in high grain production [60]. Similarly, maximum HI was recorded with Kufanzik and Hirna under optimal moisture level; similarly it was also found that changing moisture from Optimal to waterlogging growth condition affected most of the tested varieties but not Hirna, suggesting that Hirna has potential to perform under flooded soil condition than the rest of varieties. The change in environmental stresses due to climate change, can reduce crop yield considerably causing low resource use efficiency [52]. These results are an important indicator that factors contributed to crop yield losses are directly cause lower values of harvest index. In our study it was observed that except Kufanzik and Hirna varieties, the occurrence of moisture stress during the growth period significantly reduced both grain yield and HI in all varieties, but the reduction was more pronounced on chercher variety under moisture deficit than waterlogging condition.

## Conclusion

Plants have a remarkable ability to alter their development in response to different environmental cues or stress, and this phenotypic plasticity allows them to continually adapt to their local environment, which is a necessity for plants as sessile organisms. However, the response of common bean varieties under changing soil moisture conditions has not been well addressed. From this study, it was observed that different common bean varieties had different responses or phenotypic plasticity under changing stress conditions.

Among the tested varieties, it was observed that the Hirna and Kufanzik varieties were considered tolerant because they had higher yields and HI than the Chercher, Awash-1, and Ado varieties. This suggested that the phenotypic plasticity to adapt to changing moisture levels and performance was higher in Hirna and Kufanzik than in the other tested varieties, and they may be the most promising for maintaining phenotypic plasticity under waterlogging and drought stress environmental conditions.

## Materials and methods

### Growth conditions and plant material

A greenhouse study was carried out from September 2019 to December 2019 at Hawassa University College of Agriculture located in Hawassa at an altitude of 1700 m.a.s.l. (7°3′N and 38°28′E for this experiment). Five common bean varieties (Chercher, Ado, Kufanzik, Awash-1 and Hirna varieties) were collected from Haramaya University, Ethiopia. The genotypes have different growth habits and have bushy and semi climbing natures (Table 9).

**Table 9:** Common bean varieties used for the experiment

N^o^	Variety	Growth habit	Pedigree	Seed color	Released by	Year released
1	Chercher	DB	ATTT-165–96	White	HU	2006
2	Ado	DSC	SAB-736	White	MARC	2014
3	Kufanzik	IC	MX-8754–9 M	Pinto	HU	2008
4	Awash-1	DSC	Extrico-23	White	MARC	1990
5	Hirna	DB	ECAB-0203	Red	HU	2012

where MARC is the Melkasa Agricultural Research Center, *HU* is Haramaya University, *DB* Determinate bush, *DSC* Determinate semi climbing, *IC* indeterminate climbing

### Experimental design and treatments

For this study, a pot experiment was used in a greenhouse, and the experiment was arranged as a completely randomized design in a factorial combination of moisture levels and common bean varieties. The combination of three moisture levels (control, waterlogging (in which soil receives more water than it can absorb) and moisture deficit) and five common bean varieties (Chercher, Ado, Kufanzik, Awash-1, and Hirna) formed fifteen treatment combinations (Table 6). For effective utilization of irrigation water and optimum growth of common bean cultivars, a 75% field capacity (FC) soil moisture level was considered [61]. The experiment was carried out in a 22 cm long and 16 cm diameter plastic pot. Each pot was filled with composite soil before common bean seeds were planted. To manage the drainage of water, three holes were uniformly used from the bottom of the pot. Actual moisture treatment was imposed at 20 days at trifoliate age.

## Properties of the experimental soil

Experimental soil samples were collected at 0–30 cm depth by auger from different spots of the trial site (Agricultural College). The composited samples were dried and ground to pass through a 0.2 mm sieve before laboratory analysis, and the samples were analyzed for parameters relevant to the study at the Hawassa soil laboratory test. Soil analysis was performed as per the normal laboratory procedure.

### Data collection and measurement

#### Leaf temperature

The daily leaf temperatures were recorded three times a day (morning (6:00 am-8:00 am), midday (12:30 pm-1:30 pm) and evening (5:30 pm-6:00 pm) on 10 randomly selected days using a handheld noncontact infrared thermometer (RAYTEK, ST60 + , Raytek Inc., Santa Cruz, USA) during the experimental period from October to January 2019. The average value of 10-day measurements for each treatment is represented in Table 1.

### Relative leaf water content

Three fully expanded leaves were collected from three representative plants, and leaf discs (9 mm in diameter) were immediately weighed (leaf fresh weight); thereafter, the samples were immediately hydrated to full turgidity for 24 h by immersion in deionized water in a closed 15-ml tube at room temperature (20°C). Afterward, surface water from hydrated samples was removed with filter tissue paper and weighed to obtain fully turgid mass (leaf turgid weight). Finally, the samples were oven-dried for 24 h at 75 °C until a constant weight was obtained (leaf dry weight). Relative water content (RWC) was calculated following the method developed by [62].

$$\text{RWC}\left(\%\right)=\left[\frac{\mathrm{Leaf}\;\mathrm{fresh}\;\mathrm{weight}-\mathrm{Leaf}\;\mathrm{dry}\;\mathrm{weight}}{\mathrm{Lea}\;\mathrm{fturgid}\;\mathrm{weight}-\mathrm{Leaf}\;\mathrm{dry}\;\mathrm{weight}}\right]\text{x}100$$

### Crop phenology

All phenological data, including days to emergence (days), days to flowering, days to pod formation, and days to physiological maturity, were recorded at different growth stages from three randomly selected plants in each treatment.

### Growth and morphological data

All growth parameters, such as plant height, leaf number and internode length, were recorded at week intervals from three randomly selected plants in each treatment. Leaf area, leaf fresh weight, leaf dry weight and root length were recorded at 30 days after the start of treatment from three randomly selected plants.

### Yield components

All yield and yield components, such as the number of seeds per plant, grain yield, ad harvest index and yield component, such as pod number per plant, were collected from five plants per experimental unit. Such data were collected at physiological maturity, when 90% of the pods had lost their green color and changed their color from green to yellow following the methodology of [32].

### Statistical analysis

Each data was subjected to statistical analysis. All collected data were elaborated statistically using analysis of variance (ANOVA) performed using the SAS software package [63]. Means were separated on the basis of Fisher’s protected LSD test *(P* ≤ 0.05).

## Data Availability

The raw data of this article will be made available by corresponding author (Amsalu Gobena roro; amsalugobenaroro@gmail.com), according to the personal requests.
